# Metal-based nanoparticles in antibacterial application in biomedical field: Current development and potential mechanisms

**DOI:** 10.1007/s10544-023-00686-8

**Published:** 2024-01-23

**Authors:** Hao Jiang, Lingzhi Li, Zhong Li, Xiang Chu

**Affiliations:** 1 Sichuan Provincial Laboratory of Orthopaedic Engineering, Department of Orthopaedics, The Affiliated Hospital of Southwest Medical University, Luzhou, 646000 Sichuan China; 2 State Key Laboratory of Trauma, Burn and Combined Injury, Department of Emergency, Daping Hospital, Army Medical University, Chongqing, 400042 China

**Keywords:** Metallic nanoparticles, Anti-bacterial therapy, Drug resistance, Nanotechnology, Biomaterials

## Abstract

The rise in drug resistance in pathogenic bacteria greatly endangers public health in the post-antibiotic era, and drug-resistant bacteria currently pose a great challenge not only to the community but also to clinical procedures, including surgery, stent implantation, organ transplantation, and other medical procedures involving any open wound and compromised human immunity. Biofilm-associated drug failure, as well as rapid resistance to last-resort antibiotics, necessitates the search for novel treatments against bacterial infection. In recent years, the flourishing development of nanotechnology has provided new insights for exploiting promising alternative therapeutics for drug-resistant bacteria. Metallic agents have been applied in antibacterial usage for several centuries, and the functional modification of metal-based biomaterials using nanotechnology has now attracted great interest in the antibacterial field, not only for their intrinsic antibacterial nature but also for their ready on-demand functionalization and enhanced interaction with bacteria, rendering them with good potential in further translation. However, the possible toxicity of MNPs to the host cells and tissue still hinders its application, and current knowledge on their interaction with cellular pathways is not enough. This review will focus on recent advances in developing metallic nanoparticles (MNPs), including silver, gold, copper, and other metallic nanoparticles, for antibacterial applications, and their potential mechanisms of interaction with pathogenic bacteria as well as hosts.

## Introduction

Since the development and application of antibiotics in the last century, bacterial infection, which was deadly in the past, has ceased to be a threat endangering human life. However, the overuse of antibiotics and the evolution of microorganisms endowed them with resistance to multiple antibiotics. For example, by inhibiting cell wall synthesis, numerous microbial strains gained resistance to penicillin, vancomycin, cephalosporins, and other medications, while mutations in DNA replication, protein synthesis, and metabolism alterations of pathogenic microbes kept them free from antibiotics (Munita and Arias 2016; Wencewicz 2019). Moreover, some bacteria have already developed multiple drug resistance (MDR) to antibiotics. According to the report of the World Health Organization (WHO), MDR has already become a global crisis, bringing the new era of post-antibiotic therapy possibly leading to more than 10 million casualties per year by 2050 if no effective measures are taken (Rabiee et al. 2022). Bacterial infection has become one of the major burdens for public health and the social economy, and novel and powerful therapeutics are in great demand for fighting drug-resistant bacterial infections.

Metal materials and metal ions have a long history of antimicrobial use, such as silver ions and copper ions. The antibacterial capacities of metallic materials were discovered centuries ago, and copper and its compounds were used for treating burns, intestinal worms, ear infections, and other hygienic applications by Greeks, Romans, Aztecs, and other ancient cultures, while silver has also been extensively used for medical applications in human history (Grass et al. 2011; Rahman et al. 2022). In modern days, nanotechnology utilizes the design of nanosized agents to optimize conventional drugs, and metals in the nanoscale have a superior peculiarity in antibacterial applications, as well as good safety. Nanoparticles (NPs) are characterized by their nanoscale size, which often exhibits at least one dimension of less than 100 nm. Compared to traditional drugs or biomaterials, NPs show excellent performance in biomedical uses, including enhanced pharmacokinetics, superb catalytic properties, on-demand release of loading agents, special surface interactions with mammalian or bacterial cells, etc., indicating an interest in antibacterial applications (Chouirfa et al. 2019; Kim et al. 2021; Souza et al. 2019). For example, numerous efforts have been made to use antibacterial NPs for surface modification by different fabrications of artificial antibacterial surfaces, such as surface functionalization, derivatization, polymerization, and mechanical architecture modification. Medical devices and implants would be able to resist the adhesion and inhibit the proliferation of bacteria, thus reducing the risk of surgery or implant-associated hospital-acquired infections (Kheiri et al. 2019; Yu et al. 2015; Swartjes et al. 2015; Lv et al. 2014).

Given that metal-based nanoparticles are characterized by a high surface-to-volume ratio, which endows them with physical and chemical properties superior to those of bulk materials, large efforts have been made to develop antibacterial metallic nanoparticles. Metal-based NPs or metallic NPs exhibit desirable physiochemical traits that favor their antibacterial ability. The antibacterial activity of MNPs is multi-dimensional, not only the released metal ions and the oxidative stress induced by MNPs would lead to microbial death, but they are also widely used for their excellent optical properties and used in photo-activated therapies, including photothermal and photodynamic therapy in antibacterial stewardship (Yougbare et al. 2021; Mutalik et al. 2022; Yougbare et al. 2020; Mutalik et al. 2022).

The utilization of the antimicrobial activity of metals by nanotechnology is currently regarded as the last-line defense against infectious diseases. Since metal-based materials at the nanoscale have the ability to target various pathogenic microbes and to compromise the development of drug resistance, they have been proven to be as effective as conventional antimicrobial agents such as antibiotics and can be active even toward multidrug-resistant strains through different mechanisms (Sanchez-Lopez et al. 2020; Lemire et al. 2013; Bottollier et al. 2020). This review focuses on recent advances in metal-based nanoparticles for the antibacterial steward, along with the current understanding of their antibacterial mechanisms as well as drawbacks in their future development.

## Overview of the antibacterial mechanisms of metal nanoparticles

**Fig. 1:**
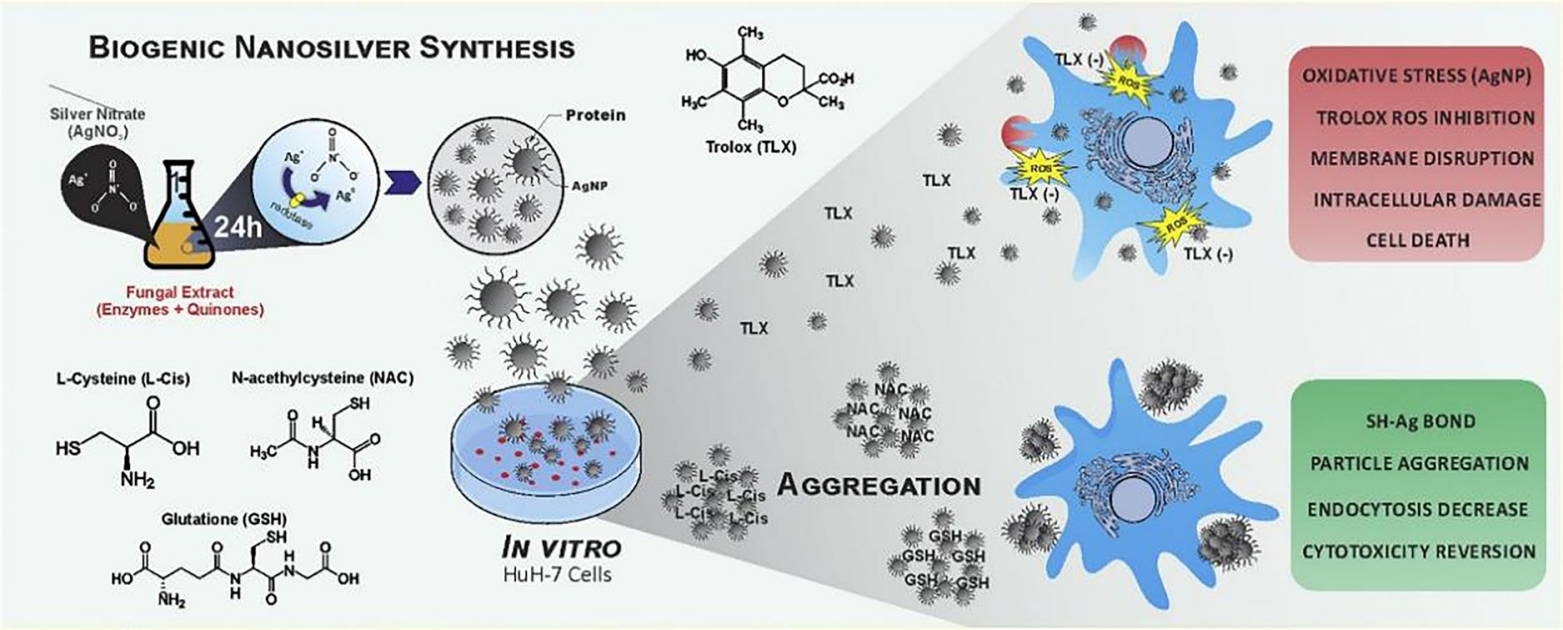
Investigation of the molecular mechanisms of AgNP cytotoxicity by using antioxidants, showing the molecular interaction between the thiol-antioxidants and non-thiol-antioxidants with chemically and biologically synthesized AgNP. Both antioxidants could mitigate ROS production in Huh-7 hepatocarcinoma cells. Nanomedicine. 2020 Feb;24:102130

In contrast to bulk materials or ionic metals, the structure, morphology, and composition of MNPs are complicated, and numerous chemical and physical properties are involved in the antibacterial toxicity of MNPs. The precise mechanisms of the antibacterial capacity of NPs have not been fully elucidated, and great efforts have been made to determine this. Generally, the current understanding of the antibacterial mechanism of MNPs is presented in Fig. 1. The bactericidal capacities of NPs are multidimensional depending on their versatile physiochemical properties. First, ionic metals released from MNPs have versatile antibacterial functions; they have potent antibacterial toxicity by interacting with the phospholipid layers in bacterial cells or interfering with the activity of various intracellular biomacromolecules, such as DNA and enzymes (Mutalik et al. 2022).

Despite the natural bactericidal features of metal materials, the nanostructures of MNPs also play an essential role in inducing antibacterial effectiveness, the cell-surface interface dynamics are one of the key mechanisms for the antibacterial effect of NPs, and the nanostructure and surface topography of NPs significantly affect their antibacterial activity. In recent decades, the main focus has been on studying the chemical reactions between NPs and bacterial cells that reveal bacterial adhesion on the biosurface (Echeverria et al. 2020). Recently, inspired by the innate bactericidal effectiveness of certain natural nanostructures, an increasing number of reports have applied the surface nano-topography effect to design novel artificial antibacterial surfaces (Song et al. 2022; T. 2020). NPs are also capable of binding to bacterial cells by electrostatic interactions and disrupting the outer membrane integrity, thus leading to the leakage of intracellular components and eventually the death of bacterial cells (Makabenta et al. 2021).

Moreover, the excitation of oxidative stress by reactive oxygen species (ROS) through introducing NPs is generally conferred as the nanotoxicity of NPs, and this feature also allows NPs to be lethal for bacterial cells. ROS are a family of contributors or inducers of oxidative stress, which includes singlet oxygen, peroxide, hydroxyl radicals, and hydrogen peroxide, leading to oxidation or peroxidation of lipids, nuclei, proteins, or other biomolecules, resulting in dysfunction of cellular processes and eventually causing bacterial death. Moreover, ROS are also regarded as one of the key reasons for NP antibacterial mechanisms; ROS can damage the cell wall, interfere with membrane permeability, and increase the proton motive force loss so that cells internalize and take up toxic NPs and their released agents (Salas-Orozco et al. 2023). Meanwhile, intracellular ROS also decreases periplasmic enzyme activity, which is essential for sustaining the physiological processes of bacterial cells.

## Metal nanoparticles with inherent antibacterial activities

### Silver nanoparticles

Among all kinds of antibacterial agents at the nanoscale, silver NPs are no doubt the most widely used agent due to their broad-spectrum bactericidal properties and robust antimicrobial efficacy against bacteria, fungi, and viruses. As a noble metal, silver is eco-friendly and biocompatible, and although the cost of silver is not very low, its nanocomposite can be readily synthesized for large-scale production (Rizzello and Pompa 2014). Considerable efforts have been devoted to understanding the antibacterial nature of silver materials, and there are two most studied theories for its antibacterial mechanisms. The first and most widely elucidated mechanism is cell disruption, which is caused by silver ions. Silver nanoparticles with a well-designed surface-to-volume ratio would be beneficial for antibacterial activity by releasing silver ions to interact with the cell membrane and penetrate bacterial cells to cause cell death by damaging proteins and breaking nuclei. The antibacterial activity of silver ions mainly depends on their chemical nature as Lewis acids; they tend to react with Lewis bases, such as phosphorous and sulfur, which constitute the majority of the cellular membrane, protein, and DNA (Tang and Zheng 2018).

Another important toxicity of silver agents is the oxidative stress caused by free radical formation, including superoxide anions, hydrogen peroxide, hydroxyl radicals, and singlet oxygen (Komazec et al. 2023; Miranda et al. 2022). Intracellular free radicals are often kept at homeostasis by cell respiration and free radical scavengers such as glutathione (GSH) under physiological conditions. Specifically, as Fig.1shows, Luiz A B Ferreira and colleagues investigated the molecular mechanisms of the cytotoxicity of AgNPs in the Huh-7 cell line. The thiol-antioxidants (N-acetyl-L-Cysteine, L-Cysteine, and glutathione) and non-thiol-antioxidants (Trolox) could alleviate the ROS generation of AgNPs in vitro experiments, showing that AgNPs can interact with sulfide, chloride, and many other chemical groups (Ferreira and Dos Reis 2020). Silver ions can also bind to the thiol group of GSH and other enzymes related to antioxidation, resulting in the accumulation of intracellular free radicals and eventually leading to cell death.

The antibacterial activities of silver NPs not only rely on silver ions but also on many aspects of physicochemical properties that affect the antibacterial effectiveness of silver NPs, including size, shape, stability, or surface modifications. The surface charge of AgNPs is one of the most important factors that affects their toxicity and bioaccumulation dynamics (Zhang et al. 2020), a recent study by Enerelt Urnukhsaikhan and collegues showed that biosynthesized AgNPs by ultilizing*Carduus crispus* showed different surface charge along with different inhibition zone in *M. luteus* and *E. coli*dishes (Urnukhsaikhan et al. 2021). In a similar report, Agnieszka Gibała explored the toxicity of AgNPs by synthesized from several biologically active compounds, and the results showed that positively charged arginine-stabilized AgNPs have most potent biocidal property (Gibala et al. 2021). The size effect is another important factor for the antibacterial property. Ekaterina A Skomorokhova et al. studied the bio activity of AgNPs with different size (10, 20, and 75 nm), and found that the smallest nanoparticle displayed the largest antibacterial activity, but similar bio-safety regarding mammalian cells (Skomorokhova et al. 2020). Despite the surface charge and size, the shape of AgNPs also matters in their antibacterial toxicity, in a recent report by Lisa M Stabryla et al., AgNPs with different shape were studied, including cube-, disc-, and pseudospherical-AgNPs, for their antibacterial activity against*E. coli*, the results showed that the antibacterial potency of AgNPs depends on their shape and varies when measured by their mass, surface area, and particle number, the disc-shaped AgNPs presented highest antibacterial activity which is evaluated by EC_50_ in *E. coli*when using a mass-based dose metric, while the cubic AgNPs is most potent when using surface area and particle number as dose metric (Stabryla et al. 2023).

Biofilm is responsible for the failure of many medical devices, including catheters and orthopedic implants, and it is a major reason for the development of drug resistance. The dense structure of biofilms endows them with enhanced tolerance to external stimuli, including host immune clearance and antimicrobial drugs. Once a biofilm is established, the host immune system becomes more vulnerable. For example, in a mature biofilm of *Staphylococcus aureus*, polymorphonuclear neutrophils can penetrate the biofilm and produce cytokines in the infectious foci; however, these attacks are not powerful enough to eradicate the microbes and may instead release cytotoxic agents and contribute to injury to the infection site, thus ultimately leading to osteolysis in some severe cases (Leid et al. 2002; Cantero et al. 2015; Wagner et al. 2003). In addition to the effectiveness of eradicating planktonic microbes, numerous studies have shown that AgNPs have superb potential in antibiofilm activity. A pioneering study on the antibiofilm usage of AgNPs was proposed in 1997 by J P Guggenbichler and colleagues, who used a polymer matrix impregnated with silver nanoparticles to prevent biofilm formation on the catheter surface and release bactericidal silver ions sustainingly (Guggenbichler et al. 1999).

The antibiofilm activity of AgNPs is multidimensional, AgNPs are capable of interacting with the cellular and extracellular nucleic acids in biofilms (Essghaier et al. 2023). Formation of reactive oxygen species (ROS) by AgNPs would cause oxidative stress and lead to unfavorable modifications in biomolecules, thus damaging the intracellular proteins, DNA, lipids, as well as extracellular EPS and DNA. To be noticed the antibiofilm efficacy also varies in AgNPs with different physiochemical properties. The size and shape affect the antimicrobial effect of AgNPs which interact with microorganism, including their disruption to the cell membrane, and the internalization by the bacterial cells. For example, in the work by Perla Alejandra Hernández-Venegas, AgNPs in 5.4 and 17.5 nm were assessed for their antimicrobial activity by using clinical isolated oral biofimls from periodontal disease and healthy participants, and the AgNP in 5.4 nm diameter showed a stronger antimicrobial activity with MIC of 71.7 ± 39.1 µg/mL, when compared to larger particles in 17.5 nm (MIC: 146.9 ± 67.3 µg/mL) (Hernandez-Venegas et al. 2023).

**Fig. 2:**
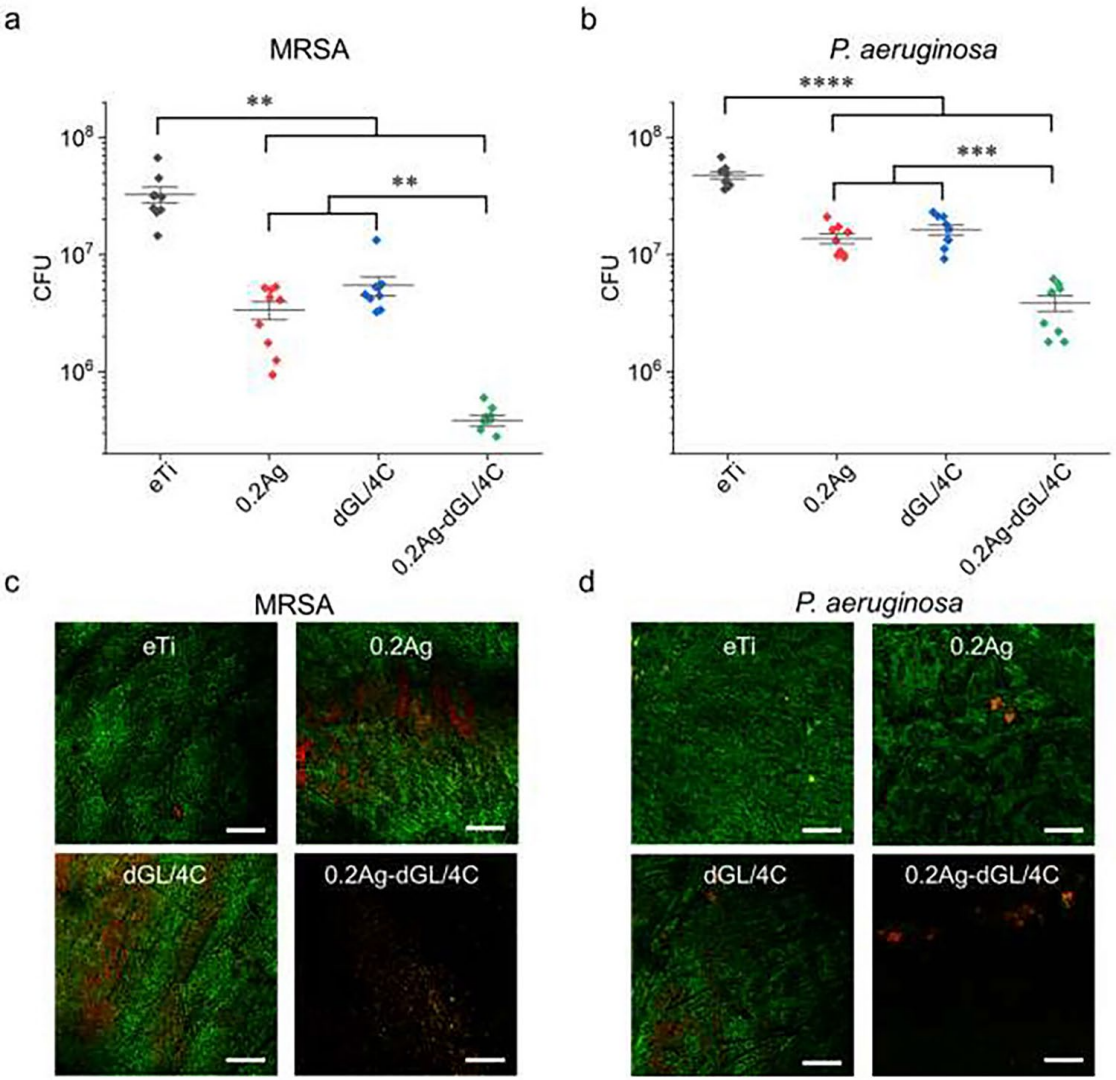
In vitro antimicrobial activity of the hybrid nanocoatings against MRSA and *P. aeruginosa*. **A**, **B**: CFU of MRSA (**A**) or P. aeruginosa (**B**) biofilms; **C**, **D**: representative LIVE/DEAD stain CLSM images of MRSA (C) or *P. aeruginosa* (D) biofilms. All biofilms were grown on the tested surfaces after 6 h incubation in quasi-static conditions with different treatments, * p-value < 0.05; ** p-value < 0.01; *** p-value < 0.001; **** p-value < 0.0001. Three biological replicates with three samples in each experiment for each group were performed for each bacteria strain (N = 9). All scale bars are 100 μm. Acta Biomater. 2022 Mar 1;140:338–349

In the past decade, AgNPs have attracted tremendous interest in clinical applications in the fields of trauma and orthopedics since perioperative infection caused by surgical implants or prostheses greatly threatens the prognosis of patients (From 2018; Kaushal et al. 2023). One of the most common applications of AgNPs is as a surface coating or outer layer of implants to reduce infection-associated surgical failure. For example, Ye Zhou and colleagues utilized a hybrid nanocoating for dental implants by combining antimicrobial peptide amphiphiles and AgNPs, and this nanocoating demonstrated remarkable antimicrobial activity against implant infection-associated pathogens both in vitro and in vivo, including*Enterococcus faecalis*, *S. gordonii*, *S. aureus*, and *P. aeruginosa*(Fig. 2) (Ye et al. 2022). Monica Thukkaram et al. applied a gas aggregation source (GAS) and a plasma-enhanced chemical vapor deposition (PE-CVD) method to achieve controllable nanocomposites of amorphous hydrocarbon and AgNPs on the surface of titanium. The nanocomposite coating provided excellent antibacterial effects and biocompatibility, while enhanced proliferation of MC3T3 was observed after application of the material (Thukkaram et al. 2020).

The successful exploration of AgNPs in this field stressed the promising prospects for further applications; however, the long-term safety and biocompatibility of AgNPs remain to be fully studied. Moreover, the interaction between AgNPs and mammalian cells, as well as the metabolism, excretion and accumulation of AgNPs in the human body, has not been well explored. Although it is promising for AgNPs to be utilized as antibacterial agents with little concern for inducing drug resistance, more efforts should still be made to translate AgNPs from bench to bedside.

### Gold nanoparticles

Ionic gold, including Au^+^ and Au^3+^, also shows high antimicrobial activity against various bacterial strains. Dasari and colleagues studied the IC_50_ of ionic gold in bacteria, and their data showed Au^+^ with an IC_50_ of 0.30–0.50 µM and Au^3+^ with an IC50 of 0.320–0.50 µM for *Salmonella typhiurium*, *S. aureus*, and *E. coli*^45^. Moreover, ionic gold exhibited a concentration-dependent effect on antibacterial activity against drug-resistant *P. aeruginosa*^*46*^. However, as the “noblest” material among metals, bulk gold is highly inert, stable, and hardly dissociates into ions. These properties allow gold to be highly biocompatible and well accepted in many biomedical applications.

Interestingly, when cut down into ultrasmall sizes, the inertness of gold materials (which become gold nanoparticles or nanoclusters) changed markedly. Gold nanoparticles exhibit distinct properties from the corresponding bulk material, and different sizes and shapes of AuNPs exhibit different physicochemical profiles, including color in solution form and surface plasmon resonance (SPR) (Amina and Guo 2020). AuNPs are capable of quenching proximate fluorophores by the deactivation pathway by generating an overlap between the SPB of AuNPs and emission spectra of fluorophores, which is called fluorescent resonance energy transfer (FRET). This property can be found in small-size AuNPs, especially that of 1 nm AuNPs (Amina and Guo 2020). Another quenching caused by AuNPs is called photoinduced electron transfer (PET), where the nanoparticles accept the photon and quench the fluorophores.

**Fig. 3:**
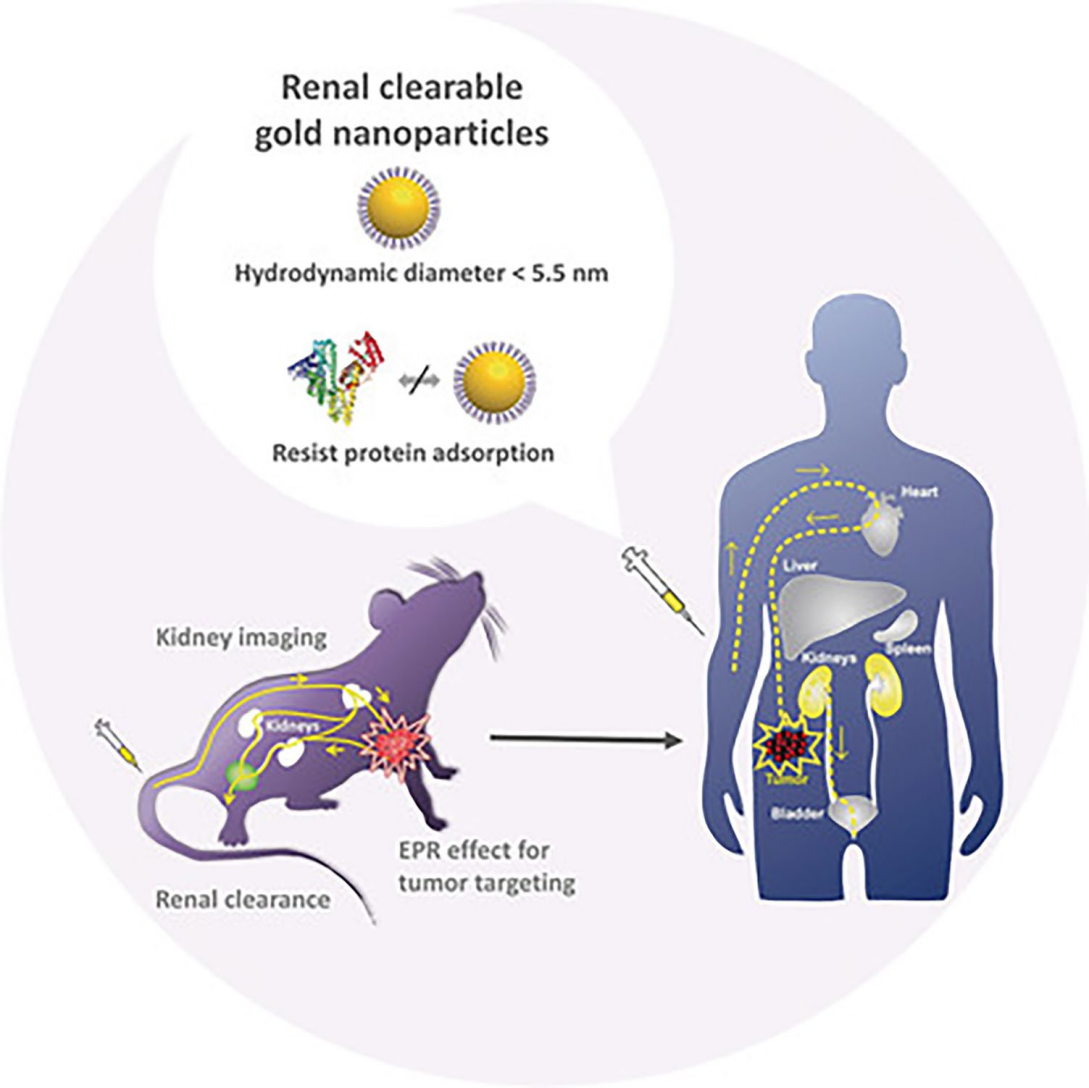
Animal studies of renal clearable gold nanoparticles from the Zheng group in the past decade are integrated with the latest FDA guidance on nanomedicines. Angew Chem Int Ed Engl. 2019 Mar 22;58 (Yougbare et al. 2021):4112–4128

For decades, AuNPs have been applied in tissue engineering, drug delivery, bacteria eradication, and many other biomedical fields due to their superb physicochemical nature. They display outstanding performance in various studies, and they are one of the most stable, safe, biocompatible, and easy-to-synthesize nanomaterials. Several publications of preclinical studies in recent years have showed that, AuNPs can be metabolized and excreted through renal clearance with minimum accumulation in the body (Yu et al. 2019; Zhou et al. 2022), rendering them with a promising potential for clinical translation (Fig.3).

The antibacterial application of AuNPs has been studied for a long time. In general, the antibacterial activity of AuNPs can be induced by causing apoptosis, DNA damage, cell membrane damage, electron transfer chain damage, ROS production, and disruption of the metabolic pathways of microbes. AuNPs can be adhere to the bacterial membrane through electrostatic reaction, and then interact with lysine on the membrane of gram-positive bacteria, leading to irreversible pores forming in the bacterial membrane, and eventually cause bacterial death, in the other hand, AuNPs can be internalized by bacterial cells to decrease intracellular ATP and disturb metabolism (X. 2022). Isabel U Foreman-Ortiz et al. studied the perturbation to phospholipid membranes induced by anionic gold nanoparticles (AuNPs), the result showed that the gramicidin A (gA) ion channels activity in the lipid bilayer were affected with the membrane integrity intact, the anionic AuNPs disrupt ion channel function by interfering the mechanical properties of the surrounding bilayer (Foreman-Ortiz et al. 2020). By utilizing the high affinity of AuNPs to biomolecules, AuNPs can easily aggregate on the cell surface, and affect the membrane permeability by causing pits, fissures or pores, in this situation, the mass of adherent AuNPs depends on the surface charge as well surface-to-volume ratio (Timoszyk and Grochowalska 2022).

Unlike AgNPs, AuNPs are not often recognized as inherent antimicrobial agents, and the antibacterial application of this type of material is usually combined with chemicals or drugs, for example, antibiotics. By functionalizing AuNPs with chemicals or drugs, the lack of specificity, insufficient cell penetration, and short half-life of the agents can be compensated and thus exhibit stronger antibacterial activity. The pioneering study on functionalizing vancomycin to AuNPs by Gu and colleagues first reported that a nanocomposite based on AuNPs exhibited desirable antibacterial activity against vancomycin-resistant *Enterococci* (Hongwei Gu et al. 2003). By copping vanillin to AuNPs (VAuNPs) to inhibit the MexAB-OprM efflux pump, the work by Sagar S Arya and colleagues showed that the MIC of seven different antibiotics against clinically isolated*P. aeruginosa*was significantly decreased and successfully reversed antimicrobial resistance in this strain (Arya et al. 2019).

### Copper nanoparticles

Copper has a long history of antimicrobial applications, and its compounds have been widely and extensively used as antifungal agents in agriculture, especially on grapes and potatoes (Ermini and Voliani 2021). Compared to silver- or gold-based materials, the cost of copper is much cheaper, copper-based materials are more easily accessible, and copper-based NPs (CuNPs) can be synthesized in an eco-friendly manner with well-tolerated biocompatibility (Mitra et al. 2020; Ingle et al. 2014). CuNPs have inherent advantages over other metallic NPs; they have cheaper and easier accessibility and can be dissolved to release free ions more quickly than noble metal-based NPs, generating higher effectiveness regarding copper ion activity in ROS generation, antimicrobial function, and chemical catalysis (Ermini and Voliani 2021). Meanwhile, copper is an essential element for cell signaling pathways and biomolecular processes. By an elaborate design of CuNPs, the released copper ions are capable of enhancing certain cellular pathways, including anti-inflammatory regulation, immune response, or tissue regeneration (Wang et al. 2021).

Due to the special properties of CuNPs, increasing attention has been given to their biomedical applications, especially their antibacterial use. The mechanisms of the antibacterial capacity of CuNPs have not yet been fully illustrated; not only are the chemical properties and concentrations important in the antibacterial performance, but the physical properties, including the shape, size, surface-to-volume ratio, and surface charge, are critical for the antimicrobial activity (Slavin et al. 2017). The major focus of research on the antibacterial mechanism of metal-based nanoparticles is ROS generation to cause oxidative stress in the bacterial cell, which then leads to loss of cellular physiological function and membrane integrity and cell death (Balderrama-Gonzalez et al. 2021; Wang et al. 2017). However, studies on the generation of ROS, integrative interaction between particle and bacterial cells, and molecular mechanism caused by CuNPs are relatively short. Meng-Jiun Lai et al. explored the antimicrobial effect of CuNPs of different sizes and concentrations, and the results showed that reactive ROS and genomic DNA damage induced by CuNPs were influenced by the size and concentration of CuNPs, and multiple mechanisms simultaneously occurred during the interaction between CuNPs and*Escherichia coli* (Lai et al. 2022).

During the process of CuNP synthesis, natural compounds are often used as stabilizers, including plant extracts and microbiome ferments. This would allow the products to be more eco-friendly and tolerant for mammalian cells; moreover, those natural compounds or natural products may serve as a synergistic effector for the antibacterial activity of CuNP (Antonio-Perez et al. 2023). For example, in the work reported by J. Bocarando‑Chacón et al., CuNPs were synthesized through green chemistry by using the extract of*Opuntia ficus-Indica* and *Geranium* as a reducing agent. They acquired spherical CuNPs of 3–10 nm for antimicrobial testing on *E. coli*, and the results showed that CuNPs of 6 nm with a concentration of 150 µg/mL had the most potent bactericidal activity (Bocarando-Chacón et al. 2020). Hongwei Zhao and colleagues used*Allium eriophyllum Boiss* leaf aqueous extract in the synthesis of CuNPs and investigated its activity against fungi and bacteria by comparison with *Allium eriophyllum Boiss* extract or CuSO_4_alone (Zhao et al. 2020). Through a macrobroth dilation test, CuNPs showed the most effective result, with minimal inhibitory concentrations against fungi (*Candida albicans*, *Candida glabrata*, *Candida guilliermondii*, *Candida krusei*), gram-negative bacteria (*Salmonella typhimurium*, *Escherichia coli*, *Pseudomonas aeruginosa*) and gram-positive bacteria (*Staphylococcus aureus*, *Streptococcus pneumoniae*, *Streptococcus pneumoniae*). Notably, the MICs of CuNPs in most species were 4 times lower than CuSO_4_ and 2 times lower than *Allium eriophyllum Boiss* extract.

However, the active nature of copper may cause toxicity to mammalian cells or lead to long-term toxicity to the human body once it accumulates in the system. Although copper is an essential and fundamental element for physiological processes in the body, it can be toxic to cells once it reaches the threshold dose. In vitro experiments have shown that CuNPs at a certain range of concentrations affect cell viability in a composition-, shape-, and size-dependent manner (Sanchez-Lopez et al. 2020). This outcome mainly resulted from oxidative stress and disturbed cellular homeostasis caused by the nanoparticle itself or released copper ions. Unlike noble metals used for antibacterial applications, copper can be oxidized more easily and tends to dissolve as ions; although this property endows copper with wide antibacterial applications, the consequent toxicity is also nonnegligible (Zou et al. 2021). This should be taken into serious consideration when designing a copper-based nanomaterial for biomedical applications, and strategies that minimize the toxicity of CuNPs are crucial. CuNPs could induce oxidative stress not only to pathogens but also to mammalian cells, In vivo experiment showed that CuNPs up-regulated the caspase 3 level and down-regulated the expression of apelin receptor in murine uterus, and induce cell apoptosis (Anima et al. 2023).

The toxicity of CuNPs can be reduced by the design of surface ligands for the NPs, which interact with environmental proteins to form a protein corona and affect their internalization by cells, recognition by the immune system, and eventually metabolism and persistence in the body (Akhuli et al. 2021; Packirisamy and Pandurangan 2022). However, an elaborate nanoparticle composition would result in more sophisticated kinetics in the body system, hampering the translation of CuNPs from the laboratory to the real world (Table 1).

**Table 1:** MNPs with inherent antibacterial activity and associated mechanisms in their toxicity and biocompatibility

Type of MNPs	Publication date	Interaction with host cells/microorganism	Involved toxicity and biocompatibility	Ref.
AgNPs	2020	Huh-7 cell (in vitro)	Induce ROS generation	(Ferreira et al. 2020)
AgNPs	2020	*C. vulgaris* (in vitro)	Surface charge-dependent antibacterial activity	(Zhang et al. 2020)
AgNPs	2021	*M. luteus*, *E. coli* (in vitro)	Surface charge-dependent antibacterial activity	(Urnukhsaikhan et al. 2021)
AgNPs	2021	*E. coli*, *S. aureus*, *Candida albicans* (in vitro)	Surface charge-dependent antibacterial activity	(Gibala et al. 2021)
AgNPs	2020	A549 cells, *E. coli* (in vitro) C57Bl/6 mice (in vivo)	Size-dependent antibacterial activity	(Skomorokhova et al. 2020)
AgNPs	2023	*E. coli* (in vitro)	Shape-dependent antibacterial activity	(Stabryla et al. 2023)
AgNPs	2023	Human derived biofilm (in vitro)	Size-dependent antibacterial activity	(Hernandez-Venegas et al. 2023)
AgNPs	2022	*Enterococcus faecalis, S. gordonii, S. aureus, and P. aeruginosa* (in vitro and in vivo)		(Ye et al. 2022)
AgNPs	2020	MC3T3 cells (in vitro) *E. coli, S. aureus* (in vitro)	Improved cell proliferation	(Thukkaram et al. 2020)
AuNPs	2021	Drug-resistant *P. aeruginosa* (in vitro)	Ionic gold	(Torres et al. 2021)
AuNPs	2022	Mice (in vivo)	Renal clearable	(Zhou et al. 2022)
AuNPs	2020	NA	Perturbation to phospholipid membranes	(Foreman-Ortiz et al. 2020)
AuNPs	2019	*P. aeruginosa* (in vitro)	Inhibit the MexAB-OprM efflux pump	(Arya et al. 2019)
CuNPs	2022	*E. coli* (in vitro)	Membrane disruption, ROS generation, DNA damage depends on size and concentration	(Lai et al. 2022)
CuNPs	2020	*E. coli* (in vitro)	Size of nanoparticle	(Bocarando-Chacón et al. 2020)
CuNPs	2020	*Candida albicans, Candida glabrata, Candida guilliermondii, Candida krusei, Salmonella typhimurium, Escherichia coli, Pseudomonas aeruginosa,Staphylococcus aureus, Streptococcus pneumoniae, Streptococcus pneumoniae* (in vitro)	Antioxidant wound healing	(Zhao et al. 2020)
CuNPs	2021	COV434 cells (in vitro)	Oxidative Stress via tMAPK14-Nrf2 pathway	(Zou et al. 2021)
CuNPs	2023	Mice (in vivo)	Up-regulated the caspase 3	(Anima et al. 2023)
PdNPs	2021	Multi-drug resistant *Staphylococcus aureus, Escherichia fergusonii, Acinetobacter pittii, Pseudomonas aeruginosa, Aeromonas enteropelogenes*, and *Proteus mirabilis* (in vitro)		(Sonbol et al. 2021)
PdNPs	2020	*S. aureus, S. pyrogens, B. subtilis, E. aerogenes, K. pneumoniae, P. vulgaris* (in vitro) Red blood cells (in vitro)	Anti-inflammatory activity performed with Red blood cells	(Mohana et al. 2020)
ZnO-NPs	2022	*Escherichia coli, Pseudomonas aeruginosa, Salmonella typhi, Serratia marcescens, Klebsiella pneumoniae, and Proteus mirabilis* (in vitro)	Bacterial cell membrane damage	(Krishnamoorthy et al. 2022)

### Others

In addition to Ag, Au, and Cu, which have been the metals most commonly used to fight bacterial infection since ancient times, nanotechnology also endows other metal materials with superb antibacterial activity. For example, palladium (Pd) nanoparticles, a transition metal, have gained increasing attention for applications in biological and chemical fields due to their unique electronic, optical, and photothermal properties. Hana Sonbol and colleagues reported a PdNP that synthesized by a one-step, cost-effective, and green manner by utilizing a brown alga extract and Padina boryana, and evaluated the antibacterial and antibiofilm activity of prepared PdNPs. The PdNPs exhibited potent antibacterial and antibiofilm property against several drug-resistant strains, including multi-drug resistant *Staphylococcus aureus*, *Escherichia fergusonii*, *Acinetobacter pittii*, *Pseudomonas aeruginosa*, *Aeromonas enteropelogenes*, and *Proteus mirabilis*, with MIC varies from 62.5 to 125 µg/mL (Sonbol et al. 2021). Similarly, S. Mohana and colleagues reported PdNPs with sizes from 13 to 18 nm, which exhibited higher antimicrobial effects against gram-positive strains (*S. aureus, S. pyrogens, B. subtilis*) than gram-negative strains (*E. aerogenes, K. pneumoniae, P. vulgaris*), and they exerted anti-inflammatory activity on red blood cells (Mohana and Sumathi 2020). The antibacterial activity of PdNPs is usually thought to be related to the generation of oxidative stress.

**Fig. 4:**
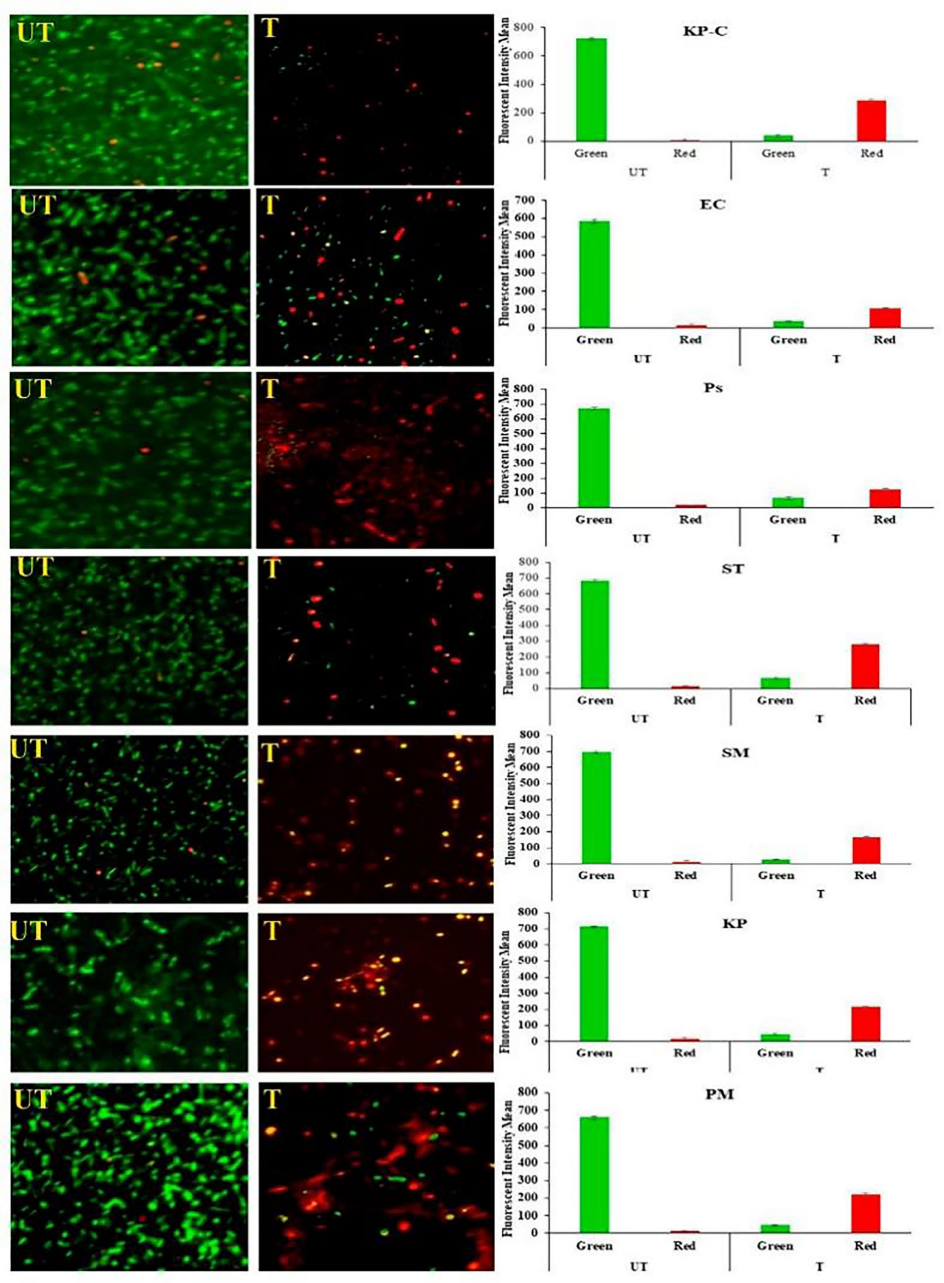
The double staining(SYTO9 and PI) assay for membrane damage of E. coli, K. pneumoniae, P. aeruginosa, S. typhi, S. marcescens, and P. mirabilis treated by ZnO NPs. Live bacteria with intact membranes appear green, and the injured/damaged bacterial cells appear yellow/red. The bar diagram represents the fluorescent mean intensity. Copyright (2022) authors. Licensee MDPI, Basel, Switzerland, Molecules 2022, 27 (Souza et al. 2019), 2489

Zinc oxide nanoparticles (ZnO-NPs) are another promising antimicrobial agent in the postbiotics era due to their excellent properties, including stability, biocompatibility, high activity and chemical, electrical and magnetic properties (Mutukwa et al. 2022). In addition, ZnO-NPs are safe and barely induce bacterial resistance. The antibacterial effectiveness of ZnO-NPs is multidimensional; it can cause oxidation of bacterial cells by the generation of hydrogen peroxide, and the binding of the material itself on the cell surface of bacteria would lead to electrostatic force, which would eventually cause membrane disruption and death of bacterial cells. In a recent work by Rajapandiyan Krishnamoorthy and colleagues, ZnO-NPs demonstrated broad-spectrum effectiveness against β-lactamase-producing bacteria due to increased ROS and malondialdehyde caused by the NPs, with MICs ranging from 0.04 to 0.08 mg/mL and minimal lethal doses of 0.12 to 0.24 mg/mL, Fig.4shows the bacterial cell membrane would be damaged due to ZnO NPs treatment (R. 2022). Notably, there are still unknown antibacterial mechanisms in these metal-based NPs. The studies on ZnO- or Pd-based NPs are not as extensive as those on Ag, Au, or Cu, and the main focus on the interaction of microorganisms and NPs lies on the surface interaction or the oxidative stress induced by NPs, probably because NPs are not always internalized by microorganisms and interfere with their intracellular physiological processes. Once the material itself and the released ions pass through the cell membrane and enter the cytoplasm of bacterial cells, the interaction of NPs and bacteria might be multidimensional, and future efforts could be made to explain the detailed mechanism of these antibacterial NPs.

## Metal nanoparticles as excellent booster for antibiotics

Many pathogenic bacteria have evolved the capability to evade the host immune defense to survive, and intracellular bacteria make it difficult for the host immune system to clear these pathogens and to make it difficult for antibiotics or other antimicrobial agents to achieve infection eradication due to the limitations in the physicochemical properties of currently available antibiotics, such as low cellular permeability, poor retention and stability in mammalian cells (Tian et al. 2022; Subramaniam et al. 2021). Fortunately, nanotechnology provided an alternative for a better way of delivering antibacterial agents into the desirable site, both for extracellular and intracellular pathogenic microorganisms.

Past years have witnessed the flourishing development of nanotechnology due to its promising applications in many biomedical fields, including drug delivery, gene therapy, tissue engineering and theranostics. In addition to the excellent antibacterial nature of some metal-based nanoparticles, these materials are capable of being utilized as delivery vehicles for a wide range of therapeutic agents. Conventional antibacterial drugs demonstrate limitations that greatly hamper their applications in the post-antibiotic era. Although antibiotics, AMPs, and other antimicrobial agents have shown high effectiveness against bacteria in vitro, the therapeutic effect may be limited due to their low bioavailability, short-term body clearance to maintain an effective blood concentration, or insufficient drug concentration in infection foci due to biofilm formation (Canaparo et al. 2019). Nanotechnology provides a solution for the abovementioned limitations. Nanoparticles can serve as remarkable vehicles for the delivery of antimicrobial agents to infectious sites by bacteria-specific targeting, lead to a long-term blood concentration of antimicrobial agents, release the drug load in a controllable manner by tactful manufacture, and even reverse drug resistance in some strains.

NPs are readily functionalized in numerous methods when designing drug delivery nanoplatforms. Noncovalent binding is often used for loading drugs on NPs. To this end, drug release does not require any specific bond cleavage to provide efficient drug release, and changes in intrinsic physical forces are sufficient for this purpose. Moreover, therapeutic agents, targeting agents, and other functionalization agents can also be bonded to NPs through covalent binding; in this way, NPs are more similar to prodrugs, which would be delivered to the desired site and release the drug payload by either external or internal stimuli (Amina and Guo 2020).

**Fig. 5:**
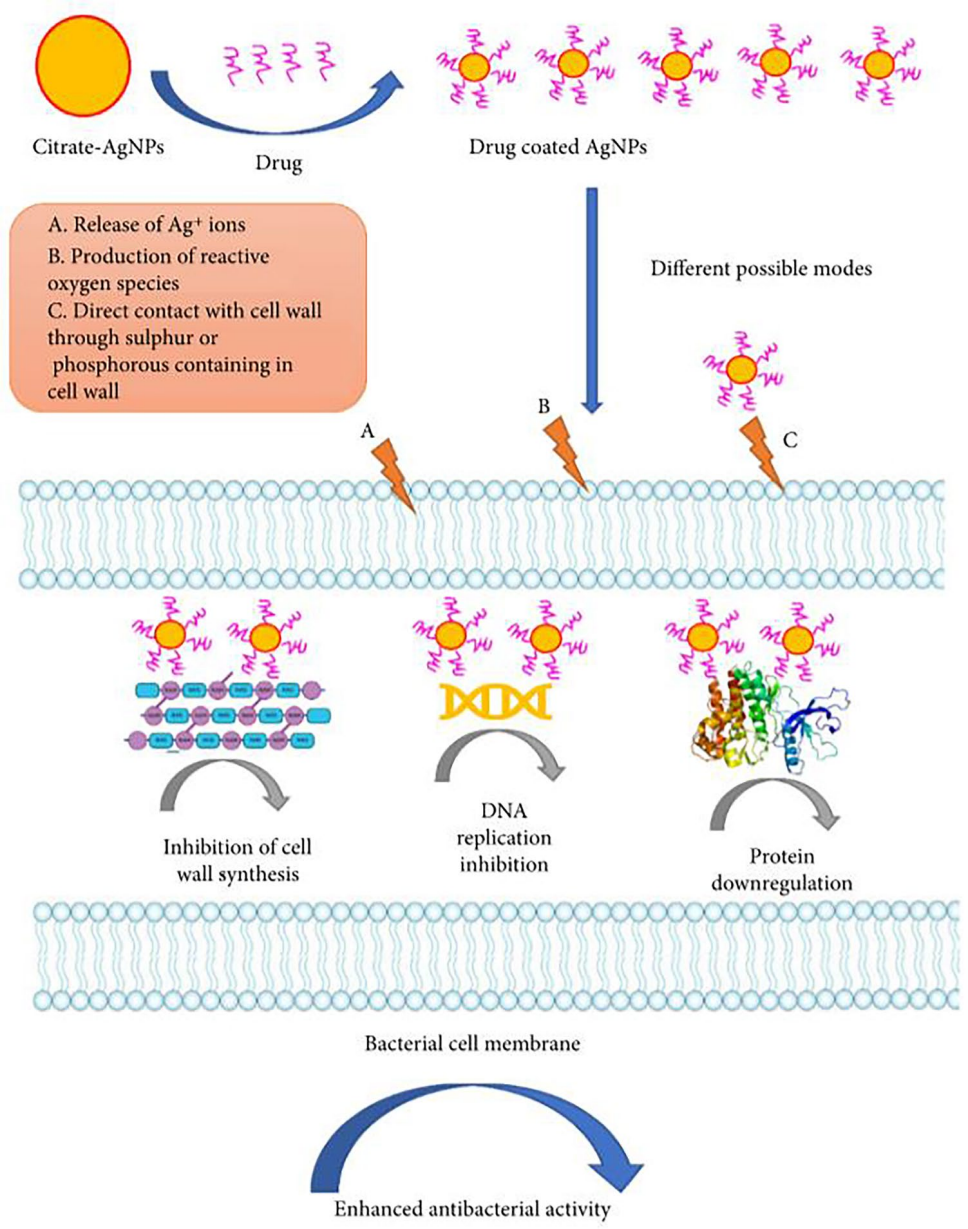
The proposed antibacterial mechanisms of vancomycin-coated AgNPs. Copyright © 2022 Mohsina Patwekar et al., Biomed Res Int. 2022 Aug 21;2022:3682757

By using metallic nanoparticles as functional carriers for drug payload, the bactericidal activity of various antibiotics can be magnified significantly. Turki Al Hagbani and colleagues adopted vancomycin in AuNPs through a facile one-pot method to obtain V-GNPs, and in vitro antibacterial experiments showed that V-GNPs have much more effective antibacterial activity than vancomycin against several strains (Hagbani et al. 2022). Specifically, the inhibitory efficacy of V-GNPs was 1.4-fold higher in*Escherichia coli*, 1.6-fold higher in *Klebsiella oxytoca*, 1.8-fold higher in *Pseudomonas aeruginosa*, and 1.6-fold higher in *Staphylococcus aureus*. Similarly, as Fig. 5illustrated, the work by Mohsina Patwekar et al. showed that the antimicrobial activity of vancomycin could be enhanced by doping on the AgNP nanoplatform, so synthesized Van@AgNPs boosted the antibacterial activity of vancomycin against gram-positive strains as well as gram-negative strains (Patwekar et al. 2022). Peng yang et al. prepared ultrasmall AuNPs within 2–3 nm for the delivery of cationic metallopolymer PCo(cobaltocenium homopolymer) and penicillin-G bioconjugates, thus obtaining antimicrobial Au@PCo-Peni nanoparticles (Yang et al. 2019). By utilizing the large surface of NPs to attach bacterial cells along with the cationic charged NPs for preferred interaction with negatively charged bacteria, the activity of Au@PCo-Peni was boosted remarkably, and it showed a broad-spectrum antimicrobial effect on both gram-positive and gram-negative bacteria, with enhanced antimicrobial activity compared to the conventional form of penicillin-G as well as PCo-penicillin bioconjugates. Nadia Ghaffar et al. reported a green synthesis method based on*Ricinus communis*leaf extracts for preparing several types of metallic nanoparticles, including AgNPs, ZnO NPs, CuO NPs and FeO NPs, as nanoplatforms to conjugate streptomycin (Ghaffar et al. 2022). Among these NPs, ZnO NPs showed the highest amplifying effect on enhancing streptomycin activity against*S. aureus.* Afrah Nawaz et al. reported ciprofloxacin-loaded AuNPs (CIP-AuNPs) for treating *Enterococcus faecalis*-induced infection in the liver and kidneys in a murine model. In vivo investigation showed that CIP-AuNPs provided an enhanced anti-infection effect, while the hemolytic characteristics of ciprofloxacin were significantly limited due to the negative charge on the nanoparticle surface and the reduced dose of free CIP in the physiological environment (Nawaz et al. 2021).

In general, the current literature and recent advances in metallic nanoparticles and antibiotic nanocomposites show that the former is a potent booster for the former in many ways, including improving the pharmacokinetic behavior, thereby reducing undemanding side effects and enhancing the antimicrobial activity, and even providing a stronger efficacy against biofilm-associated infection. However, most publications in this field are focused on the enhancement of the antimicrobial activity of antibiotics when conjugated with nanoparticles; however, the underlying mechanism has not been fully understood or interpreted thoroughly. There are several generally recognized mechanisms involving the enhancement of metallic nanoparticles in the antibacterial effectiveness of antibiotics: (1) metal-based nanoparticles are capable of neutralizing the negative charge of bacterial membranes, therefore damaging the permeability of bacteria; (2) the ROS generated by most metal-based nanoparticles damage the bacterial membrane or interfere with the antioxidant balance in bacterial cells; and (3) some metal-based nanoparticles tend to interact with intracellular biomolecules in bacteria, including DNA and proteins, and disrupt their tolerance to external stimuli as well as the potential for developing resistance to antibiotics. Notably, the interaction of MNP-antibiotic composites and bacterial cells is complicated; not only does the size, shape, surface charge, or other physiochemical profile of MNPs affect the physiological process of bacteria, but the release behavior of metal ions and free antibiotics, as well as their synergistic effects, are also important for antimicrobial activity. To understand the specific antibacterial enhancement mechanism, these relevant aspects need to be taken into account.

## Future perspectives of MNPs in clinical translation for antibacterial application

The crisis of drug-resistant infection is rising, and both scientific and industrial interests are now focusing on developing novel strategies for antimicrobial agents with high potential for clinical translation. As ancient anti-infection agents, metallic materials have been applied for centuries; currently, the rapid development of nanotechnology renders metallic materials with better physiochemical properties in the biomedical field. There is no doubt that MNPs are one of the most potent and promising antibacterial agents in current days, with the high effectiveness of their inherent antimicrobial activity and the boosting effect of MNPs on various antibiotics. However, certain concerns still exist for the clinical translation of MNPs.

Since metallic materials do not tend to form nanostructures by self-assembly, complicated and sophisticated methodologies are necessary for metallic materials to transform into nanoformulations. For MNP fabrication, many physicochemical methods are applied in industry, including chemical reduction, chemical solution deposition, photochemical reduction, electrochemical reduction, and other methods. These methodologies introduce reactive and toxic agents for reducing and stabilizing MNPs; if not purified and handled outrightly, considerable toxicity would also be introduced in vivo as well as to the environment (Gour and Jain 2019).

In recent decades, green chemistry has been developed to synthesize MNPs from metal salts by exploiting biologically active compounds as novel reducing agents, thus providing more eco-friendly and biocompatible alternatives for synthesizing MNPs (Jain et al. 2021). Recently, various microorganisms and plants have been employed for the green synthesis of MNPs, including AuNPs, AgNPs, zinc, and palladium NPs. Specifically, microbial nanofactories using bacteria, algae, yeast, fungi, and many other marine organisms and phototrophic eukaryotes showed excellent efficacy in synthesizing MNPs in an eco-friendly manner. Moreover, many other natural products have also been applied to replace conventional reducing chemistry; for example, soluble starch and maltose were used as stabilizing agents in AgNP synthesis (Sarkar et al. 2021; Passlick et al. 2010). These methodologies provide higher stability and biocompatibility of MNPs for biomedical use, and they shed light on a novel green industry for MNP manufacture, putting forward the clinical translation of MNPs.

Another key issue that should be taken into consideration is the off-target effect of MNPs in vivo. The higher biocompatibility and extended pharmaceutical half-life of MNPs indeed provide better antibacterial activity because effective agents are delivered to the infectious foci more efficiently, but it might cause some undemanding outcomes for the body since MNPs accumulate in other organs. To this end, rationally designed infection-specific targeting strategies are essential to reduce off-target effects, and a better understanding of the mechanisms of pharmaceutical behavior, in vivo toxicology, and the interaction of MNPs with different types of mammalian cells are of utmost importance in improving the safety of MNPs in biomedical applications.

Due to the complicated formulation of MNPs, comprehensive molecular signaling and intracellular mechanisms are involved in the interaction between host cells as MNPs. The toxicity of MNPs to tissues and organs is complicated and depends on the physiochemical characteristics and the dissolution and aggregation behavior of MNPs under different physiological conditions. Current knowledge is still unable to elucidate the unknown effect and mechanism of nanotoxicity of MNPs. Recently, high-throughput sequencing technologies have been extensively developed, including RNA-seq and proteomics, and even integrated multiomics technology, which would help researchers gain a deeper and wider understanding of the host-MNP interaction and better elucidate the biological effects induced by NPs in multiple dimensions. Understanding the complicated interaction between MNPs and host cells is still the top priority for advancing the application and translation of MNPs.
